# Impact of diabetes on long-term survival in elderly liver cancer patients: A retrospective study

**DOI:** 10.1515/med-2024-1096

**Published:** 2025-01-10

**Authors:** Dan Chen, Xiaoxiao Gao, Yaqing Wang

**Affiliations:** Health Management Center, The Fourth Affiliated Hospital of Nanjing Medical University, Nanjing, Jiangsu, 210000, China; Department of Endocrinology, The Fourth Affiliated Hospital of Nanjing Medical University, Nanjing, Jiangsu, 210000, China

**Keywords:** diabetes, liver cancer, elderly, prognosis

## Abstract

**Background and aim:**

Liver cancer is a prevalent and life-threatening condition, particularly among elderly individuals. The association between diabetes, a chronic metabolic disorder, and the onset and advancement of liver cancer has been widely acknowledged. However, the effect of diabetes on the survival of older patients with liver cancer has been a topic of debate. In light of this, we undertook a retrospective study to assess the impact of diabetes on the overall survival (OS) of elderly individuals diagnosed with liver cancer.

**Methods:**

In this retrospective analysis, we examined clinical data from liver cancer patients aged 80 years or older who underwent diagnosis and treatment at a solitary medical center from January 2010 to December 2019. Comprehensive records encompassing baseline information, treatment protocols, diabetes history, and mortality during follow-up were meticulously documented. Employing the Kaplan–Meier method and the Cox proportional hazards model, we sought to assess the influence of diabetes on both the OS and recurrence-free survival (RFS) in elderly individuals diagnosed with liver cancer.

**Results:**

This study comprised 244 elderly liver cancer patients, with 68 individuals reporting a history of diabetes. In the unadjusted Kaplan–Meier survival analysis, the diabetes group exhibited a lower OS compared to the non-diabetes group. Utilizing a multivariate Cox proportional hazards model, diabetes emerged as a prognostic factor influencing OS (hazard ratio, HR = 1.782 [1.163–2.743], *P* = 0.043). Regarding RFS, unadjusted Kaplan–Meier analysis revealed a diminished RFS in the diabetes group compared to the non-diabetes group. In the multivariate Cox proportional hazards model, diabetes remained a significant prognostic factor impacting RFS (HR = 1.742 [1.083–1.546], *P* = 0.041).

**Conclusion:**

Our study indicates a significant impact of diabetes on both OS and RFS among elderly liver cancer patients. These insights may contribute to more precise guidance and recommendations for the treatment of this specific demographic, offering valuable information for healthcare practitioners working with elderly individuals diagnosed with liver cancer.

## 1 Introduction

Liver cancer poses a significant global public health challenge [1], with an estimated 841,000 new cases and 782,000 deaths reported in 2020 [2,3]. The incidence of liver cancer rises with age, and elderly individuals face an elevated risk of developing this condition, attributed to the cumulative impact of environmental and genetic risk factors [4]. Moreover, diabetes, characterized by persistent high blood glucose levels, has been recognized as an independent risk factor for liver cancer [5,6].

The correlation between diabetes and liver cancer among elderly patients has been extensively explored in recent years. Multiple studies have indicated that diabetes is linked to an elevated incidence and increased mortality of liver cancer in the elderly population [7,8]. A meta-analysis of 26 cohort studies revealed a 43% increase in the risk of liver cancer associated with diabetes in the general population [9]. Additionally, an independent study highlighted that diabetes was specifically correlated with a higher risk of liver cancer mortality in elderly men [10]. Furthermore, a retrospective study demonstrated a significant association between diabetes and compromised overall survival (OS) as well as disease-free survival in elderly patients diagnosed with hepatitis B virus-related hepatocellular carcinoma (HCC) [11] Concurrently, Kramer and Natarajan [12] emphasized the importance of adequate glycemic control, concluding that reasonable glycemic control could reduce the risk of HCC by 31% in patients with nonalcoholic fatty liver disease (NAFLD).

Nevertheless, not all studies have consistently observed a significant association between diabetes and the survival outcomes in elderly patients with liver cancer. A substantial cohort study, encompassing over 10,000 patients, reported no noteworthy difference in OS between individuals with and without diabetes who were diagnosed with liver cancer. Similarly, a retrospective study found no association between diabetes and prognosis in elderly patients specifically with HCC.

As a result, the impact of diabetes on the survival of elderly patients with HCC and the extent of this impact remain subjects of controversy. In an effort to contribute clarity to this issue, we conducted a retrospective study to evaluate the influence of diabetes on the survival of elderly patients with liver cancer. Our findings aim to offer more precise guidance and recommendations for the treatment of elderly individuals grappling with liver cancer.

## 2 Materials and methods

### 2.1 Patient selection

This retrospective study encompassed all elderly patients aged 80 years or older diagnosed with liver cancer and treated at the Department of Hepatobiliary Surgery, a single medical center in China, during the period from January 2010 to December 2019. To ensure data integrity, patients with incomplete clinical information or those who had initiated treatment before their initial hospital visit were excluded from the study.

The diagnostic criteria for type 2 diabetes, following the guidelines of the World Health Organization and International Diabetes Federation, required meeting one of the following conditions: a fasting plasma glucose level ≥ 7.0 mmol/L (126 mg/dL); a random plasma glucose level ≥ 11.1 mmol/L (200 mg/dL) with diabetes symptoms such as polyuria, polydipsia, or fatigue; or a 2-h plasma glucose level ≥ 11.1 mmol/L (200 mg/dL) during a 75 g oral glucose tolerance test.

Strict inclusion criteria were applied: (1) patients aged 80 years or older at the time of diagnosis; (2) confirmed diagnosis of primary liver cancer through pathological examination or radiological findings; and (3) initial detection of liver cancer. Exclusion criteria comprised: (1) incomplete clinical data, including missing laboratory results or imaging studies; (2) previous liver cancer treatment before the initial hospital visit; and (3) diagnosis of other malignancies before or after liver cancer.

Approval for this retrospective study was obtained from the Ethics Committee of The Fourth Affiliated Hospital of Nanjing Medical University, adhering to the principles outlined in the Declaration of Helsinki. Informed consent was provided by all patients. Imaging examinations, such as computed tomography (CT)/magnetic resonance imaging (MRI), were conducted, and liver cancer diagnosis was confirmed by two experienced radiologists. Surgical treatment cases were pathologically verified for liver cancer based on postoperative pathology.

### 2.2 Follow-up

All patients were subject to continuous follow-up until either death or the conclusion of the study on February 28, 2023. Follow-up appointments were scheduled every 3 months for the initial year post-diagnosis, transitioning to every 6 months thereafter. During each follow-up visit, a comprehensive assessment was conducted, encompassing physical examinations, blood tests (including liver function tests and alpha-fetoprotein [AFP] levels), and imaging studies such as ultrasound, CT, MRI, and positron emission tomography, as deemed necessary. This rigorous follow-up protocol ensured thorough monitoring and evaluation of each patient’s health status throughout the study duration.

### 2.3 Clinical data collection

From electronic medical records, we retrospectively gathered the following data: demographic characteristics (age, sex), diabetes status, liver function test results (including alanine transaminase [ALT], aspartate transaminase [AST], albumin [ALB], total bilirubin [TBIL], and prothrombin time [PT], serum AFP levels), tumor stage (as per the Barcelona Clinic Liver Cancer staging system), treatment regimens (covering surgical resection, radiofrequency ablation, transcatheter arterial chemoembolization, and supportive care), and mortality recorded during the follow-up period. Diabetes status was confirmed based on a documented history of diabetes or the use of hypoglycemic agents. This comprehensive dataset allowed for a thorough analysis of various factors influencing the outcomes of elderly patients with liver cancer.

### 2.4 Data analysis

Continuous variables, assuming a normal distribution, are expressed as mean value ± standard deviation, and differences between them are assessed using the *T*-test. Categorical variables are compared between two groups using either the Chi-square test or Fisher’s exact test. For the generation of OS and recurrence-free survival (RFS) curves, the Kaplan–Meier method was employed, with survival curve comparisons executed using the log-rank test. To evaluate the impact of diabetes on OS and PFS while adjusting for potential confounding factors such as age, sex, AFP levels, tumor stage, treatment regimens, and liver function tests, the Cox proportional hazards model was applied.

All statistical analyses were conducted using SPSS 25.0 (IBM, Armonk, New York, USA), with a significance level set at *P* < 0.05 (two sides). Graphical representations were created using R language (version 4.0.5) and GraphPad Prism (version: 8.0). Sample size estimation was performed using PASS (version: 11.0) before the initiation of the study.


**Ethics approval and consent to participate:** This research was performed in accordance with the Declaration of Helsinki and was approved by the Ethics Committee of The Fourth Affiliated Hospital of Nanjing Medical University. All patients provided informed consent.
**Informed consent:** Written informed consent for publication was obtained.

## 3 Results

### 3.1 Baseline information of liver cancer patients with and without diabetes

**Figure 1:**
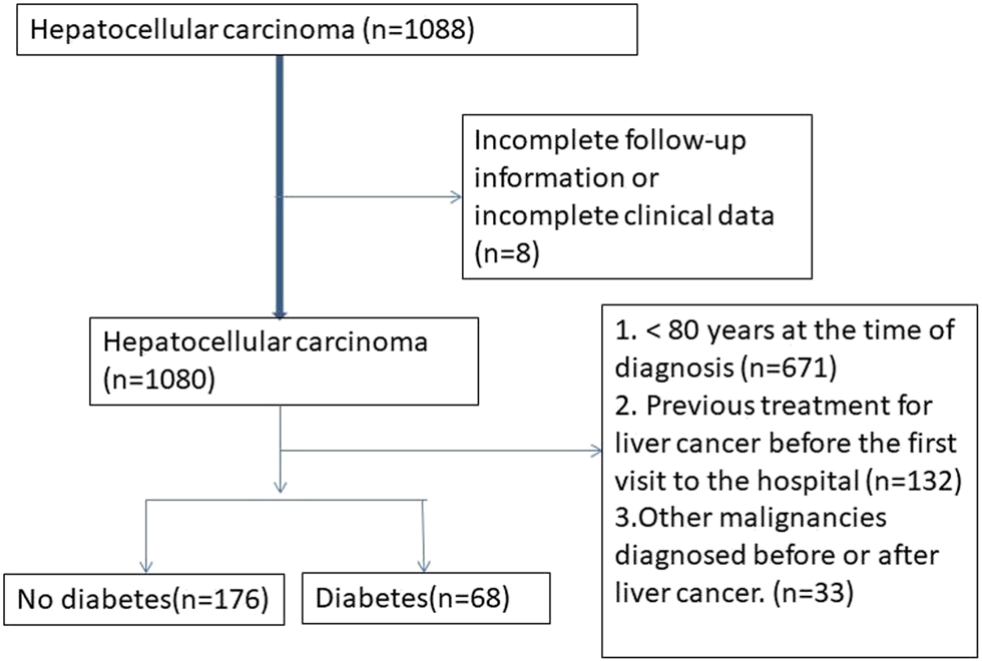
Inclusion and exclusion table for HCC patients.

The inclusion and exclusion criteria for patients are comprehensively outlined in Figure 1. Patients with diabetes exhibited a mean age of 84 ± 3.2 years, which was significantly higher than their non-diabetic counterparts (82 ± 4.0 years, *P* = 0.032). Both groups displayed a similar gender distribution, with 54.4% of diabetic patients being male and 60.8% of non-diabetic patients being male (*P* = 0.402). The majority of patients in both groups had cirrhosis (82.4% of diabetic patients and 84.1% of non-diabetic patients), with no significant difference observed between the two groups (*P* = 0.758). Similar Eastern Cooperative Oncology Group (ECOG) performance status was noted in both groups, with 86.8% of diabetic patients and 84.1% of non-diabetic patients having an ECOG score of 0–1 (*P* = 0.758).

Patients with diabetes exhibited slightly higher levels of ALT (36.8 ± 10.5 U/L) compared to those without diabetes (34.2 ± 8.6 U/L, *P* = 0.103). However, there was no significant difference in AST levels between the two groups (*P* = 0.387). Non-diabetic patients demonstrated higher levels of serum ALB (42.5 ± 3.2 g/L) compared to diabetic patients (40.8 ± 3.8 g/L, *P* = 0.012). No significant differences were observed in TBIL or PT levels between the two groups (*P* = 0.412 and *P* = 0.246, respectively). The proportion of Hepatitis B virus (HBV) and Hepatitis C virus (HCV) infections did not differ statistically between the two groups. The serum AFP level of diabetic patients was essentially similar to that of non-diabetic patients (*P* = 0.053), and among diabetic patients, 31 (45.6%) had AFP levels exceeding 400 ng/mL (Table 1).

**Table 1:** Baseline characteristics of patients with gastric cancer undergoing gastrectomy in the training and validation cohorts (*n* = 244)

		Diabetes (*n* = 68)	No diabetes (*n* = 176)	*P*-value
Gender (%)				0.402
	Male	37 (54.4)	107 (60.8)	
	Female	31 (45.6)	69 (39.2)	
Age (years)		82 ± 3.2	80 ± 4.0	0.032
ASA (%)				0.307
	I	217 (88.6)	94 (84.7)	
	II	28 (11.4)	17 (15.3)	
ECOG PS (%)				0.758
	0–1	59 (86.8)	148 (84.1)	
	2–3	12 (17.6)	28 (15.9)	
ALT	U/L	36.8 ± 10.5	34.2 ± 8.6	0.103
AST	U/L	30.3 ± 7.3	29.1 ± 6.2	0.387
ALB	g/L	40.8 ± 3.8	42.5 ± 3.2	0.012
TBIL	μmol/L	14.7 ± 3.3	14.3 ± 3.1	0.412
PT	s	16.1 ± 1.9	15.9 ± 1.7	0.246
AFP (%)				0.053
	≤400 ng/mL	37 (54.4)	120 (68.2)	
	>400 ng/mL	31 (45.6)	56 (31.8)	
Cirrhosis status				0.758
	Yes	56 (82.4)	148 (84.1)	
	No	12 (17.6)	28 (15.9)	
HBV				1.000
	Yes	60 (88.2)	155 (88.1)	
	No	8 (11.8)	21 (11.9)	
HCV				1.000
	Yes	3 (4.4)	7 (4.0)	
	No	65 (95.6)	169 (96.0)	
BCLC stage (%)				0.826
	A	46 (67.6)	118 (67.0)	
	B	12 (17.6)	36 (15.3)	
	C	10 (14.7)	22 (18.0)	
Tumor size (%)				0.565
	<5 cm	36 (52.9)	102 (58.0)	
	≥5 cm	32 (47.1)	74 (42.0)	
Tumor number (%)				1.000
	Single	46 (64.7)	118 (67.0)	
	Multiple	22 (35.3)	58 (33.0)	
Treatment regimens (%)				0.697
	Resection	20 (29.4)	56 (31.8)	
	RFA	16 (23.5)	40 (22.7)	
	TACE	24 (35.3)	60 (34.1)	
	Supportive care	8 (11.8)	20 (11.4)	
Fasting plasma glucose level	mmol/L	9.3 ± 2.1	5.1 ± 0.9	<0.001
Random plasma glucose level	mmol/L	11.1 ± 2.2	6.2 ± 0.8	<0.001
2-h plasma glucose level	mmol/L	15.2 ± 2.8	7.4 ± 1.4	<0.001

SD: standard deviation; ALT: alanine transaminase; AST: aspartate transaminase; ALB: albumin; TBIL: total bilirubin; PT: prothrombin time; AFP: alpha-fetoprotein; IQR: interquartile range; RFA: radiofrequency ablation; TACE: transcatheter arterial chemoembolization; ECOG: Eastern Cooperative Oncology Group.

### 3.2 Univariate and multivariate Cox proportional hazard regression analyses of OS in liver cancer patients

In the univariate analysis, male gender (HR = 1.211, *P* = 0.028), cirrhosis status (HR = 1.433, *P* = 0.014), ECOG performance status (HR = 1.925, *P* = 0.003), diabetes status (HR = 3.220, *P* < 0.001), higher levels of ALT (HR = 1.012, *P* = 0.024), larger tumor size (HR = 1.538, *P* < 0.001), multiple tumors (HR = 2.034, *P* < 0.001), and later tumor stage (HR = 1.271, *P* = 0.024) were associated with worse OS. However, in the multivariate analysis, adjusting for other covariates revealed that only tumor size (HR = 1.423, *P* < 0.001), number of tumors (HR = 1.813, *P* < 0.001), diabetes status (HR = 1.782, *P* = 0.043), and treatment regimen (resection/supportive care: HR = 0.617, 95% CI = 0.388–0.972, *P* = 0.038) remained significantly correlated with OS (Table 2).

**Table 2:** Univariate and multivariate analysis of OS in liver cancer patients in the whole cohort

	Univariate analysis			Multivariate analysis		
	*P*	HR	95% CI	*P*	HR	95% CI
**Gender**						
Male/female	0.028	1.211	1.031–1.423	0.726	0.920	0.651–1.297
**Age**						
Per years	0.852	0.972	0.705–1.364			
**Cirrhosis status**						
Yes/No	0.014	1.433	1.098–1.887	0.086	1.313	0.985–1.766
**ECOG PS**						
0–1/2–3	0.003	1.925	1.287–2.871	0.021	1.677	1.093–2.563
**Diabetes status**						
Yes/No	<0.001	3.220	2.352–4.441	0.043	1.782	1.163–2.743
**ALT**						
Per U/L	0.024	1.012	1.002–1.018	0.045	1.023	1.005–1.032
**AST**						
Per U/L	0.077	1.021	0.988–1.042			
**ALB**						
Per g/L	0.102	0.792	0.618–1.132			
**TBIL**						
Per μmol/L	0.029	1.012	1.004–1.023	0.267	1.012	0.991–1.027
**Tumor size**						
≥5 cm/<5 cm	<0.001	1.538	1.274–1.846	<0.001	1.423	1.161–1.745
**Tumor number**						
Multiple/Single	<0.001	2.034	1.492–2.778	<0.001	1.813	1.302–2.524
**Tumor stage**						
BCLC B/BCLC A	0.024	1.271	1.031–1.567	0.073	1.213	0.987–1.492
**Treatment regimens**	0.006			0.038		
Resection/supportive care		0.551	0.361–0.843		0.617	0.388–0.972
RFA/supportive care		0.682	0.441–1.062			

ALT: alanine transaminase; AST: aspartate transaminase; ALB: albumin; TBIL: total bilirubin; AFP: alpha-fetoprotein; RFA: postoperative adjuvant radiofrequency ablation; TACE: postoperative adjuvant transcatheter arterial chemoembolization; ECOG: Eastern Cooperative Oncology Group.

### 3.3 Univariate and multivariate Cox proportional hazard regression analyses of RFS in liver cancer patients who underwent radical treatment

The univariate analysis indicates that cirrhosis status (HR = 1.389, *P* = 0.023), diabetes status (HR = 3.192, *P* < 0.001), larger tumor size (HR = 1.475, *P* < 0.001), multiple tumors (HR = 2.153, *P* < 0.001), later tumor stage (HR = 1.301, *P* = 0.013), and treatment regimen (resection/radiofrequency ablation [RFA]: HR = 0.788, 95% CI = 0.534–0.910, *P* = 0.006) were significantly associated with RFS. In the multivariate analysis, after adjusting for other covariates, only cirrhosis status (HR = 1.441, 95% CI = 1.058–1.741, *P* = 0.132), diabetes status (HR = 1.742, 95% CI = 1.083–1.546, *P* = 0.041), tumor size (HR = 1.318, 95% CI = 1.073–1.618, *P* = 0.033), tumor number (HR = 2.431, 95% CI = 1.812–3.170, *P* = 0.041), tumor stage (HR = 1.416, 95% CI = 1.178–1.862, *P* = 0.019), and treatment regimen (HR = 0.865, 95% CI = 0.613–0.943, *P* = 0.016) remained significantly correlated with RFS. Patients with cirrhosis or diabetes faced a higher risk of recurrence, while those with larger tumor sizes, multiple tumors, and later tumor stage were also associated with an elevated risk of recurrence. Notably, patients who underwent resection exhibited better RFS outcomes compared to those who received RFA (Table 3).

**Table 3:** Univariate and multivariate analysis of RFS in liver cancer patients with radical treatment (surgical and RFA) in the whole cohort

	Univariate analysis			Multivariate analysis		
	*P*	HR	95% CI	*P*	HR	95% CI
**Gender**						
Male/female	0.113	1.105	0.881–1.343			
**Age**						
Per years	0.718	0.873	0.648–1.271			
**Cirrhosis status**						
Yes/No	0.023	1.389	1.134–1.912	0.132	1.441	1.058–1.741
**ECOG PS**						
0–1/2–3	0.138	1.742	0.868–2.472			
**Diabetes status**						
Yes/No	<0.001	3.192	2.034–5.031	0.041	1.742	1.083–1.546
**ALT**						
Per U/L	0.121	1.008	0.972–1.142			
**AST**						
Per U/L	0.065	1.017	0.989–1.134			
**ALB**						
Per g/L	0.213	0.812	0.712–1.488			
**TBIL**						
Per μmol/L	0.210	1.078	0.852–1.166			
**Tumor size**						
≥5 cm/<5 cm	<0.001	1.475	1.246–1.952	0.033	1.318	1.073–1.618
**Tumor number**						
Multiple/single	<0.001	2.153	1.774–2.913	0.041	2.431	1.812–3.170
**Tumor stage**						
BCLC B/BCLC A	0.013	1.301	1.078–1.611	0.019	1.416	1.178–1.862
**Treatment regimens**						
Resection/RFA	0.006	0.788	0.534–0.910	0.016	0.865	0.613–.943

ALT: alanine transaminase; AST: aspartate transaminase; ALB: albumin; TBIL: total bilirubin; AFP: alpha-fetoprotein; RFA: postoperative adjuvant radiofrequency ablation; TACE: postoperative adjuvant transcatheter arterial chemoembolization; ECOG: Eastern Cooperative Oncology Group.

### 3.4 Complications in HCC patients in the diabetic group vs the non-diabetic group

During the follow-up period, in the diabetic group, there were six deaths (8.8% of total patients) in the diabetic group, while there were five deaths (2.8% of total patients) in the non-diabetic group. The difference between the two groups was not statistically significant (*P* = 0.077).

In addition, liver failure occurred in three cases (4.4% of total patients) in the diabetic group, while only two cases (1.1% of total patients) occurred in the non-diabetic group. Lung infection occurred in five cases (7.4% of total patients) in the diabetic group, while only two cases (1.1% of total patients) occurred in the non-diabetic group. These differences were statistically significant between the two groups (*P* < 0.05).

Furthermore, with regard to distant metastasis of HCC, two cases (2.9% of total patients) were observed in the diabetic group, compared to one case (0.6% of total patients) in the non-diabetic group. However, there were no significant differences between the two groups in terms of other complications such as hydrocephalus, cardiovascular, and cerebrovascular events (*P* > 0.05) (Table 4).

**Table 4:** Complications during hospitalization in patients with hepatocellular carcinoma in the diabetic group vs the non-diabetic group

	Diabetes (*n* = 68)	No diabetes (*n* = 176)	*P*-value§
Number of deaths, *n* (%)†	6 (8.8)	5 (2.8)	0.077
Liver failure, *n* (%)	3 (4.4)	2 (1.1)	0.134
Hydrocephalus, *n* (%)	2 (2.9)	1 (0.6)	0.189
Lung infection, *n* (%)	5 (7.4)	2 (1.1)	0.019
Distant metastasis of liver cancer*, *n* (%)	2 (2.9)	1 (0.6)	0.189
Cardiovascular accidents, *n* (%)	1 (1.5)	1 (0.6)	0.481
Cerebrovascular accidents, *n* (%)	1 (1.5)	1 (0.6)	0.481
Tumor recurrence, *n* (%)‡	51 (75.0)	105 (59.7)	0.026

†Patient died within 90 days after admission. *After radical treatment. ‡Patients with recurrence by the time of the last follow-up. §Using the chi-square test or Fisher’s exact probability test.

### 3.5 Prognosis of elderly HCC patients in the group with diabetes vs the group without diabetes

In terms of OS, HCC patients with type 2 diabetes exhibited OS rates at 1, 3, and 5 years of 63.9, 16.7, and 0.0%, respectively, with a median survival time of 22.0 months. Conversely, for HCC patients without type 2 diabetes, the OS rates at 1, 3, and 5 years were 100.0, 78.1, and 61.5%, respectively, with a median survival time of 84.0 months.

**Figure 2:**
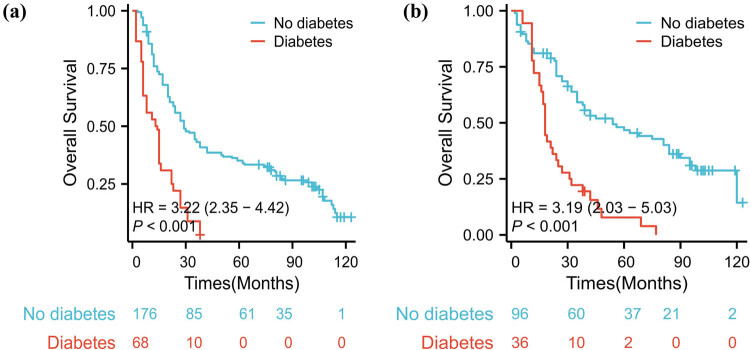
Prognostic survival of elderly HCC patients in the diabetic and non-diabetic groups. (a) represents OS for all patients and (b) represents RFS for patients receiving radical treatment.

For HCC patients who underwent radical treatment, the 1-, 3-, and 5-year relapse-free survival rates for those with and without diabetes were 69.4, 7.1, and 0.0%, and 81.1, 55.5, and 40.0%, respectively (Figure 2).

### 3.6 Efficacy of different glucose metrics to predict 5-year survival

**Figure 3:**
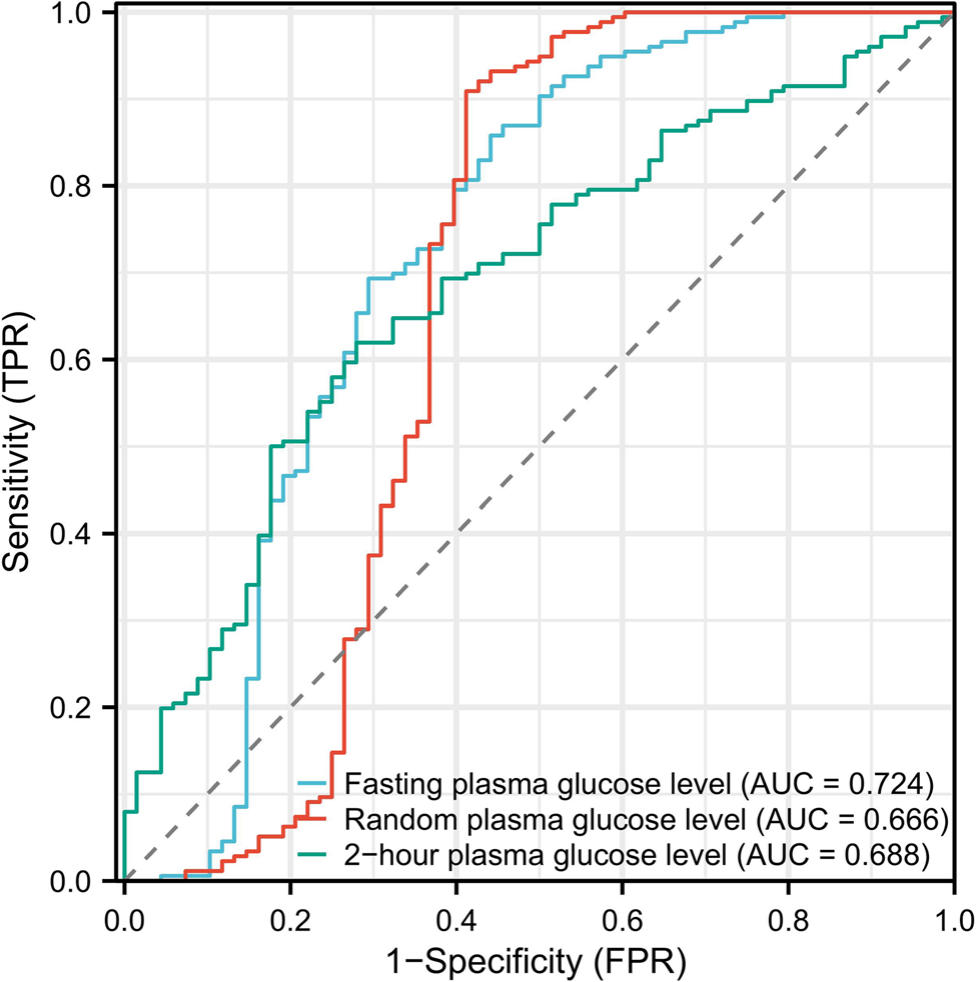
Fasting plasma glucose level, random plasma glucose level, and 2-h plasma glucose level of the ROC.

Receiver operating characteristic (ROC) curves were employed to illustrate the predictive efficacy of various glucose metrics for elderly HCC patients. The ROC curves for fasting plasma glucose level, random plasma glucose level, and 2 h plasma glucose level were plotted, where the horizontal axis represents 1-specificity and the vertical axis denotes sensitivity. The area under the curve (AUC) was determined to assess the discriminatory power of each metric. Specifically, the AUC values were 0.724 for fasting plasma glucose level, 0.666 for random plasma glucose level, and 0.688 for 2 h plasma glucose level (Figure 3).

## 4 Discussion

HCC represents a significant and escalating public health concern, marked by a rise in both incidence and mortality rates on a global scale. This concerning trend is primarily attributed to human activities that have modified the environment and lifestyle, fostering the accumulation of HCC risk factors. These risk factors encompass unhealthy dietary habits, excessive alcohol consumption, viral infections, genetic predispositions, and metabolic abnormalities [13,14]. The elderly population faces a heightened susceptibility to HCC compared to other age groups, primarily due to prolonged exposure to HCC risk factors such as persistent alcohol consumption or past hepatitis virus infections. Additionally, the gradual decline in organ function, a weakened immune system, and diminished cellular repair capabilities in the elderly contribute to accelerated physical degeneration and damage, rendering them more prone to the development of HCC.

Recent studies have emphasized that diabetes mellitus (DM) stands as one of the risk factors for HCC in the elderly [15]. With over 400 million individuals worldwide affected by DM, these patients face an elevated risk of various metabolic disorders, including liver diseases and HCC. As highlighted by Onikanni and colleagues [16], a connection exists between Type 2 DM (T2DM) and liver cancer. However, the underlying pathological and physiological mechanisms linking T2DM to HCC are multifaceted, involving associations with various conditions such as NAFLD, heightened hepatic insulin resistance, hyperinsulinemia, activation of pro-inflammatory mediators, oxidative stress, JNK-1 activation, increased IGF-1 activity, alterations in gut microbiota, and immune regulation [17,18]. Significantly, the robust association between DM and HCC extends not only to the elderly population but also to the general population. These findings underscore the necessity for heightened attention to the impact of DM on the occurrence and progression of HCC in the elderly, given their elevated incidence of DM and prolonged exposure to HCC risk factors.

Our study underscores the significant impact of T2DM on the long-term prognosis of elderly liver cancer patients, elevating both their risk of mortality and recurrence. Bragg’s prospective investigation involving 510,000 individuals across ten regions in China revealed an association between diabetes and liver cancer mortality (RR = 1.54 [95% CI, 1.28–1.86]). Similarly, Onikanni et al. [16] analyzed data from 97 prospective studies, encompassing 820,900 individuals globally, and identified a moderate association between diabetes (vs non-diabetes) and deaths related to liver, pancreatic, ovarian, colorectal, lung, stomach, and breast cancers – consistent with our study’s findings. In our investigation, T2DM exhibited a stronger correlation with OS in elderly HCC patients (HR = 3.220 [2.352–4.441]). Therefore, HCC emerges not only as a significant cause of mortality in diabetic patients but also as a noteworthy comorbidity that cannot be overlooked. Consequently, there is a call for enhanced interventions in clinical practice for elderly liver cancer patients, including the implementation of routine blood glucose-lowering therapy.

Diabetes is also a factor in the progression of liver disease [19]. Angulo et al. [20] concluded that diabetes is a significant predictor of severe liver fibrosis, while the risk of causing liver disease to progress to HCC increases with increasing duration of diabetes, with a multivariate HR of 2.96 (95% CI, 1.57–5.60) for HCC with a duration of diabetes < 2 years, 6.08 (95% CI, 2.96–12.50) < 10 years and 7.52 (95% CI, 3.88–14.58) for patients with DM ≥ 10 years, so the risk of HCC development in patients with cirrhosis in the presence of long-term DM should be tested with emphasis.

Our study has certain limitations, first, the study is a retrospective study, so selection bias and confounding bias are inevitable; second, the duration of illness of the diabetic patients we included was not discussed in subgroups, and further discussion on the duration of illness in type 2 diabetic patients should be conducted in the future; and finally, the sample size should be further expanded and multicenter data should be added to improve the external applicability of the findings.

## 5 Conclusion

Our study highlights the considerable impact of diabetes on OS and RFS in elderly patients with liver cancer. These findings have the potential to offer more precise guidance and recommendations for the treatment of elderly patients grappling with liver cancer. Moreover, the diabetic group exhibited a higher incidence of complications, underscoring the importance of directing attention toward elderly HCC patients with diabetes.
